# Exploring the origins of a signaling pathway

**DOI:** 10.7554/eLife.108735

**Published:** 2025-09-18

**Authors:** Balaji Santhanam

**Affiliations:** 1 Center of Excellence for Data-Driven Discovery, Department of Structural Biology, St Jude Children’s Research Hospital Memphis United States

**Keywords:** Wnt signaling, evolution, immunity, lipid metabolism, peptidoglycan, Other, None

## Abstract

The 19 Wnt proteins found in humans are part of a larger superfamily of proteins that are also found in Archaea and Bacteria.

**Related research article** Burroughs AM, Nicastro GG, Aravind L. 2025. The lipocone superfamily, a unifying theme in metabolism of lipids, peptidoglycan and exopolysaccharides, inter-organismal conflicts and immunity. *eLife* **14**:RP108061. doi: 10.7554/eLife.108061.

Wnt signaling pathways have many important roles in cells and are highly conserved across the animal kingdom from fruit flies to humans. In addition to Wnt signaling being involved in many processes during development, including the differentiation, proliferation and migration of cells, disruption of this pathway can lead to cancer, diabetes and other medical conditions.

The canonical Wnt signaling pathway is activated when a Wnt protein binds to a Frizzled receptor and its co-receptor (Figure 1A; Nusse and Clevers, 2017). This interaction results in the recruitment of a cytoplasmic protein called Dishevelled which, in turn, disrupts the activity of a complex that destroys β-catenin (Qin et al., 2024). This allows β-catenin to accumulate in the cytoplasm and translocate into the nucleus, where it binds to specific transcription factors and regulates the process of gene transcription.

**Figure 1.**
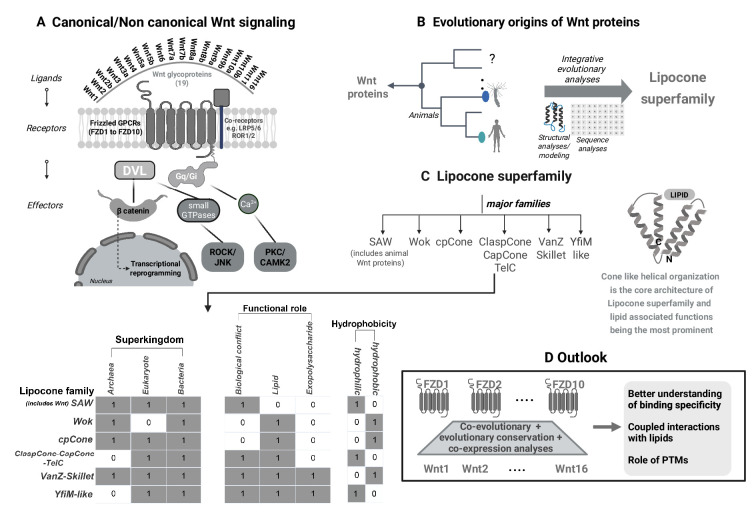
Wnt signaling and the Lipocone superfamily of Wnt proteins. (**A**) Wnt signaling involves a Wnt protein binding to a membrane-bound Frizzled receptor and its co-receptor, followed by the recruitment of a Dishevelled (DVL) protein. The canonical Wnt signaling pathway also involves β-catenin (see main text), whereas the noncanonical planar cell polarity pathway involves various small GTPases and kinases (such as ROCK and JNK), while different kinases (PKC and CAMK2) have a role in the noncanonical Wnt/calcium pathway. The noncanonical pathways also involve additional G proteins (Gq/Gi). (**B**) Burroughs et al. used a combination of techniques to explore the origins of the 19 Wnt proteins found in humans. They found that these proteins – and related proteins found in primitive animals, such as placozoans (*Trichoplax adhaerens*) and sea anemones (*Nematostella vectensis*) – were part of a larger superfamily, which they called the Lipocone superfamily. (**C**) Lipocone proteins contain four helices, which adopt a cone-like shape, and are organized into six families: SAW, Wok, CpCone, ClaspCone-CaspCone-TelC, VanZ-Skillet, and YfiM-like. Burroughs et al. then examined the phylogenetic profiles of the six families, their functional roles, and whether they were hydrophobic or hydrophilic. As discussed in the main text, three of the families were present in Archaea, Eukaryotes and Bacteria, and three were present in only two of these. The functional roles studied were the immune response, lipid binding, and association with exopolysaccharide. Again, some families performed all three roles, and some performed only one or two, with lipid binding being the most widespread. Lastly, three of the families were hydrophobic and three were hydrophilic. (**D**) The approach taken in this study could be extended to better understand the interactions between the 19 Wnt proteins and the 10 Frizzled receptors in humans, including how they interact with lipids and the influence of post-translational modifications (PTMs). Created in BioRender.

Similarly, the two known noncanonical Wnt signaling pathways are activated when a Wnt protein binds to a Frizzled receptor (Wright et al., 2018), followed by the recruitment of Dishevelled. In the case of the noncanonical planar cell polarity pathway, this is followed by a series of events that regulate the cytoskeleton of the cell, while the noncanonical Wnt/calcium pathway regulates the level of calcium inside the cell (Kühl et al., 2000; Li et al., 2020; Zhan et al., 2017). In humans, there are 19 known Wnt proteins and 10 known Frizzled receptors (Figure 1A; Grätz et al., 2023).

Wnt proteins are widely distributed across metazoans (animals) and can be traced back to primitive animals such as placozoans and sea anemones. Frizzled receptors are also predominantly found in metazoans, indicating that both the ligand and receptor components of the Wnt signaling pathway are primarily restricted to multicellular animals (Holzem et al., 2024). This highlights the pathway’s important role in the evolution of multicellularity.

Frizzled receptors belong to a large family of proteins called G protein-coupled receptors (GPCRs), whose evolutionary roots can be traced back to bacterial proteins with seven transmembrane helices (Anantharaman and Aravind, 2003). However, the evolutionary origins of the Wnt proteins themselves remained unclear until 2020, when Wnt-like proteins were identified in bacteria by Max Burroughs and L Aravind at the National Institutes of Health (Burroughs and Aravind, 2020). Previously it was thought that Wnt proteins were only found in animals, but the discovery of bacterial Wnt-like proteins suggested a more ancient origin.

Now, in eLife, Burroughs, Gianlucca Nicastro and Aravind report the results of a systematic study to explore the origins of Wnt proteins in detail (Burroughs et al., 2025). The researchers adopted an integrative approach that encompasses protein sequence, structure and contextual analyses, coupled with structural modeling, to systematically uncover the evolutionary history of Wnt proteins and identify their distant homologues (Figure 1B). They were also able to deduce that these homologue proteins – many of which were poorly characterized – contained four helices, which were arranged in a cone-like shape, and were organized into six families.

Burroughs et al. then examined the phylogenetic profiles of the six families, their functional roles, and whether they were hydrophobic or hydrophilic (Figure 1C). They found that three of the six families are present in Archaea, Eukaryotes and Bacteria, and three are present in just two of these superkingdoms. The SAW family, which includes the metazoan Wnt proteins, is seen across all three superkingdoms, and all six families are present in bacteria, suggesting a potential bacterial origin, similar to the Frizzled receptors.

The three functional roles studied by the researchers were immune response, lipid binding, and association with exopolysaccharide. Again, some families performed all three roles, and some performed only one or two. Burroughs et al. also found that the most common functional role among the families was lipid binding, which suggests that this was probably the ancestral role of these proteins. This, combined with the cone-like shape of the proteins, led to the researchers naming the overarching superfamily the “Lipocone” superfamily.

One topic for future work is to characterize the Lipocone proteins involved in the immune response in prokaryotes. The integrative approach used by Burroughs et al. could also be extended to include co-evolutionary and co-expression analyses of Wnt proteins and Frizzled receptors within metazoans (Figure 1D; Marks et al., 2011; Anishchenko et al., 2017). This would enable a comprehensive exploration of the binding specificity between the 19 Wnt proteins and the 10 Frizzled receptors. Our knowledge of this specificity is limited at present, especially at the systems level, and deciphering it could have considerable biological and therapeutic implications.
